# Role of nucleosome remodeling in neurodevelopmental and intellectual disability disorders

**DOI:** 10.3389/fnbeh.2015.00100

**Published:** 2015-04-23

**Authors:** Alberto J. López, Marcelo A. Wood

**Affiliations:** Department of Neurobiology and Behavior, Center for the Neurobiology of Learning and Memory, University of California IrvineIrvine, CA, USA

**Keywords:** epigenetics, nucleosome remodeling, autism spectrum disorders (ASD), intellectual disability, BAF53b, rubinstein-taybi syndrome, coffin-siris syndrome, nicolaides-baraitser syndrome

## Abstract

It is becoming increasingly important to understand how epigenetic mechanisms control gene expression during neurodevelopment. Two epigenetic mechanisms that have received considerable attention are DNA methylation and histone acetylation. Human exome sequencing and genome-wide association studies have linked several neurobiological disorders to genes whose products actively regulate DNA methylation and histone acetylation. More recently, a third major epigenetic mechanism, nucleosome remodeling, has been implicated in human developmental and intellectual disability (ID) disorders. Nucleosome remodeling is driven primarily through nucleosome remodeling complexes with specialized ATP-dependent enzymes. These enzymes directly interact with DNA or chromatin structure, as well as histone subunits, to restructure the shape and organization of nucleosome positioning to ultimately regulate gene expression. Of particular interest is the neuron-specific Brg1/hBrm Associated Factor (nBAF) complex. Mutations in nBAF subunit genes have so far been linked to Coffin-Siris syndrome (CSS), Nicolaides-Baraitser syndrome (NBS), schizophrenia, and Autism Spectrum Disorder (ASD). Together, these human developmental and ID disorders are powerful examples of the impact of epigenetic modulation on gene expression. This review focuses on the new and emerging role of nucleosome remodeling in neurodevelopmental and ID disorders and whether nucleosome remodeling affects gene expression required for cognition independently of its role in regulating gene expression required for development.

## Introduction

It has long been understood that gene expression is critical not only for neural development, but also for healthy cognition in the adult. The temporally and spatially specific regulation of this gene expression is critical for the aforementioned processes to occur. One basic regulatory element of gene expression is the spatial organization of DNA, extremely compacted into chromatin. Nucleosomes are the basic subunit of chromatin and consist of approximately 147 DNA base pairs spooled around a histone octamer. These nucleosomes can fluctuate between various levels of compaction, and their state is a critical and limiting factor in allowing transcription machinery to access genes of interest. Recent work has shown that various epigenetic mechanisms can regulate gene expression by altering chromatin compaction as well as providing a signal integration platform necessary to coordinate large protein complexes for transcriptional regulation. These activities have a major role in both where and when necessary genes are expressed (Barrett and Wood, 2008; Hargreaves and Crabtree, 2011; Rando and Winston, 2012).

Such epigenetic mechanisms include DNA methylation, histone modifications (including acetylation, ubiquitination, and phosphorylation), and nucleosome remodeling. The majority of attention in neuroscience has been given to histone modifications and DNA methylation as regulators of transcription. Deficits in either of the aforementioned mechanisms can have profound effects on development and adult cognition. For instance, identified mutations in methyl CpG binding protein 2 (*MECP2*) are known to cause Rett syndrome (Rett, 1966; Amir et al., 1999; Trappe et al., 2001; Guy et al., 2007; McGraw et al., 2011; Chao and Zoghbi, 2012; Katz et al., 2012; Heckman et al., 2014), whereas mutations in CREB binding protein (*CBP*) cause Rubinstein-Taybi syndrome. (Rubinstein and Taybi, 1963; Padfield et al., 1968; Hennekam et al., 1992; Petrif et al., 1995; Cantani and Gagliesi, 1998; Alarcón et al., 2004; Verhoeven et al., 2009; Wang et al., 2010; Suzuki et al., 2013; Park et al., 2014). Yet, nucleosome remodeling remains a relatively unexplored mechanism of neural epigenetics.

Nucleosome remodeling is driven by nucleosome remodeling complexes (NRCs). These nuclear enzyme complexes are capable of actively sliding, ejecting, or completely restructuring nucleosome structure (Workman and Kingston, 1998; Li et al., 2007). As such, NRCs, specifically the neuron-specific Brg1/hBrm Associated Factor (nBAF) complex, can have profound effects on neuron-specific gene expression throughout development and adulthood. Recently, human exome sequencing studies have linked mutations in BAF complex genes to intellectual disability (ID) disorders and Autism Spectrum Disorder (ASD), increasing the significance and importance of understanding the dynamics of nBAF gene regulation (Halgren et al., 2012; Santen et al., 2012a, 2013; Tsurusaki et al., 2012; Van Houdt et al., 2012; Parikshak et al., 2013; Helsmoortel et al., 2014; Miyake et al., 2014; Vandeweyer et al., 2014). This review will focus on the role of nBAF in gene regulation throughout development and adult neural function.

It is important to distinguish nucleosome remodeling from other forms of epigenetic mechanisms. Nucleosome (chromatin) remodeling specifically refers to the ATP-dependent enzymatic complexes (e.g., nBAF, SWI/SNF, INO80, ISWI, NURD) that are involved in nucleosome mobility underlying transcriptional regulation. In neuroscience however, the term chromatin remodeling is misused as a catch-all phrase (Barrett and Wood, 2008). Chromatin remodeling is distinct from chromatin modification, which refers to histone modification (histone acetylation, phosphorylation, methylation, etc). Chromatin modification has two primary functions including the regulation of DNA-histone interaction as well as serving as a signal transduction integration platform for coordinate gene regulation (Barrett and Wood, 2008).

## Nucleosome remodeling in eukaryotic development

Nucleosome remodeling, a well-studied mechanism in yeast genetics and cancer biology, is relatively uninvestigated in neuroscience. Nucleosome remodeling is driven primarily through ATP-dependent NRCs capable of altering nucleosome structure by repositioning nucleosomes along chromosomal DNA, ejecting histones, or enabling histone variants to be interchanged within the DNA/protein interactions (Varga-weisz, 2001; Teif and Rippe, 2009; Hargreaves and Crabtree, 2011; Vogel-Ciernia and Wood, 2014). Although a core ATPase enzyme is common to all complexes, the known NRCs are unique in their subunit composition, catalytic domains, complex function, and recruited proteins. There are various families of these protein complexes, including the well-studied NuRD and SWI/SNF complexes.

The SWI/SNF complex is characterized by the well conserved DNA-dependent ATPase domain and the single bromodomain; this bromodomain is known to interact with acetylated histones and stabilize SWI/SNF-histone interactions (Hassan et al., 2001; Rando and Winston, 2012). These SWI/SNF-histone interactions are key in enabling the complex to drive nucleosome remodeling. The most commonly proposed mechanisms by which SWI/SNF is believed to remodel nucleosomes is referred to as the “DNA looping” or “reptation” model (Whitehouse et al., 1999; Van Holde and Yager, 2003). It proposes that SWI/SNF breaks histone-DNA interactions to form a micro-DNA loop using a torsional domain. This loop is forced to travel the length of DNA along the nucleosome by a tracking domain in the SWI/SNF complex which causes the nucleosome to slide as a result. (Havas et al., 2000; Becker and Hörz, 2002; Zhang et al., 2006; Zofall et al., 2006; Tang et al., 2010). Additionally, SWI/SNF complexes, in conjunction with histone chaperone proteins, are known to eject histones completely from nucleosome complexes (Boeger et al., 2003; Lorch et al., 2006; Tang et al., 2010).

The SWI/SNF complex has been shown to regulate transcription in a relatively large percentage of yeast genes. Several of the genes that fall under SWI/SNF regulation are critical for M-phase transcription (Krebs et al., 2000; Rando and Winston, 2012). For example, the HO endonuclease induces yeast mating-type switching through a double strand break. It has been shown that expression of the *Ho* gene is dependent on SWI proteins 1-6 for activation (Haber and Garvik, 1977; Stern et al., 1984; Breeden and Nasmyth, 1987; Nasmyth and Shore, 2011). Elegant genetic studies have shown that SWI/SNF interacts with histone acetyltransferase (HAT) enzymes to regulate gene expression including the *Ho* gene. For example, when *Snf2* mutations are introduced to *Gcn5* mutants (a HAT enzyme in the SAGA HAT complex), offspring are developmentally inviable (Pollard and Peterson, 1997; Rando and Winston, 2012). Additionally, SWI/SNF activity shares gene targets with SAGA HAT activity, including *Ho*. SWI/SNF and GCN5 are activated during the latter stages of mitosis, where SWI/SNF is recruited to the *Ho* promoter prior to, and is required for, GCN5 HAT activity (Cosma et al., 1999; Krebs et al., 1999, 2000; Varga-weisz, 2001; Mitra et al., 2006). Thus, SWI/SNF regulation of chromatin compaction states produce temporally selective gene expression profiles that function as switches for the developing and reproducing yeast. Understanding that these mechanisms (such as histone modifications and nucleosome remodeling) do not occur in isolation from, but rather in conjunction with, each other is a fundamental principle to guide further research in dynamic gene regulation.

For example, the combinatorial activity of HATs and NRCs strongly suggests that both histone modification and chromatin remodeling are necessary for proper genetic regulation to occur. The only known NRC to have both deacetylase and ATP-dependent nucleosome remodeling is the Nucleosome Remodeling Deacetylase (NuRD) complex (Xue et al., 1998; Denslow and Wade, 2007; Zhang and Li, 2010). The Mi-2/NuRD complex is composed of seven protein subunits: HDAC1, HDAC2 (histone deacetylases), RbAp46, RbAp48 (responsible for histone-binding), an MTA protein, MBD, and a CHD (Zhang et al., 1999; Ahringer, 2000). The ATPase functions of the NuRD complex are specifically engaged when the complex interacts with chromatin, and are inactive when exposed to isolated DNA or histones (Wade et al., 1998; Brehm et al., 2000; Wang and Zhang, 2001). It is likely that NuRD-mediated nucleosome remodeling is similar to that observed with Mi-2 alone, whose ATPase activity is DNA- and nucleosome-stimulated (Brehm et al., 2000; Wang and Zhang, 2001; Becker and Hörz, 2002). This ATPase activity has been shown to promote nucleosome sliding (Aoyagi et al., 2003; Lusser et al., 2005; Bao and Shen, 2007). Moreover, MBD subunits of the NuRD complex are capable of recognizing and binding methylated DNA (Hendrich and Bird, 1998; Wade et al., 1999; Zhang et al., 1999; Bowen et al., 2004). This strongly suggests that MBD allows the NuRD complex to maintain a level of gene repression on gene targets already tagged for transcriptional silencing.

Similar to other NRCs, NuRD complexes have a vital role in regulating gene expression with temporal precision. For example, the drosophila homolog, dMi-2, has been shown to interact with the DNA-binding protein Hb to maintain repression of specific Hox genes. Double mutant animals of *Hb* and *dMi-2* show loss of Hox gene repression (Kehle et al., 1998). Moreover, recruitment of NuRD to chromatin is dependent on transcription factor binding in Drosophila (Reddy et al., 2010), specifically TTK69, which is considered to be a transcriptional repressor.

The role of NuRD throughout development is also observed in mammals. Homozygous deletion of *Mbd3* in mice results in embryonic lethality, while *M*bd2^−/−^ mice show failed gene repression (Hendrich et al., 2001). The NuRD complex is also highly expressed throughout mammalian cell types, where it also has been shown to have gene specificity, rather than functioning as a global gene repressor (Kaji et al., 2006; McDonel et al., 2009). For example, *in vitro Mbd*^−/−^ cell lines show halted differentiation. Other NuRD core subunits were shown to be reduced, such as MTA1 and MTA2, and were no longer co-precipitated from nuclear extracts. This suggests that MBD3 is a necessary component for NuRD complex stabilization. Interestingly, only a limited pool of genes were shown to be misregulated in these cells lacking *Mbd3*, particularly *Pramel7* and *Pramel6* (Kaji et al., 2006). The NuRD complex is also known to selectively target the *Htra1* promoter and deacetylate H3K27 at this promoter. Specifically, this complex has been shown to bind directly to particular histones in a trimethylated state, including H3K4me3 and H3K27me3. ES cells lacking *Mbd3* show decreased trimethylation and increased acetylated H3K27 compared to wild type cells, providing further evidence that NuRD functions to maintain specific gene repression (Reynolds et al., 2011). NuRD subunits are critical in the adult as well. Loss of NuRD related genes, particularly *Hdac1, Mta3, Chd3*, or *Chd4*, leads to chromatin defects similar to those observed throughout aging (Pegoraro et al., 2009). Further evidence suggesting NuRD is able to coordinately regulate histone modification patterns (e.g., histone deacetylation and histone demethylation) with nucleosome remodeling comes from the relatively new discovery that lysine-specific demethylase (LSD1) and JARID1B are subunits of the NuRD complex (Wang et al., 2009; Li et al., 2011). LSD1 is a demethylase that removes mono- and di-methyl groups from H3 and H4 and may work synergistically with HDAC1/2 to generate or maintain a repressive chromatin environment (Shi et al., 2005). JARID1B is a histone demethylase that also targets H3 (H3K4 more specifically) and may work in a serial manner with LSD1 in the regulation of histone demethylation (Li et al., 2011). These subunits of the NuRD complex highlight how subunit specific composition may give rise to complex interactions between nucleosome remodeling activity and histone modification activity to coordinately regulate gene expression.

A common element of NRCs is that their activity is not instructive, but rather establishes either permissive or restrictive environments for developmental gene expression. It has been suggested that NuRD complexes are critical in maintaining pluripotency in embryonic stem cells (Crook et al., 2006). The repressive role of NuRD may be critical in maintaining a molecular brake on determinant genes. Recent work has shown that functional NuRD is necessary for suppressing *Elf5* and *Eomes*, initial trophectoderm determinant genes, in a DNA methylation-dependent manner (Latos et al., 2012). While SWI/SNF complexes seem to function as positive regulators of gene expression, NuRD complexes function to maintain selective gene repression throughout development and in the adult.

## Human exome sequencing implicates BAF-related proteins in developmental disorders

**Figure 1:**
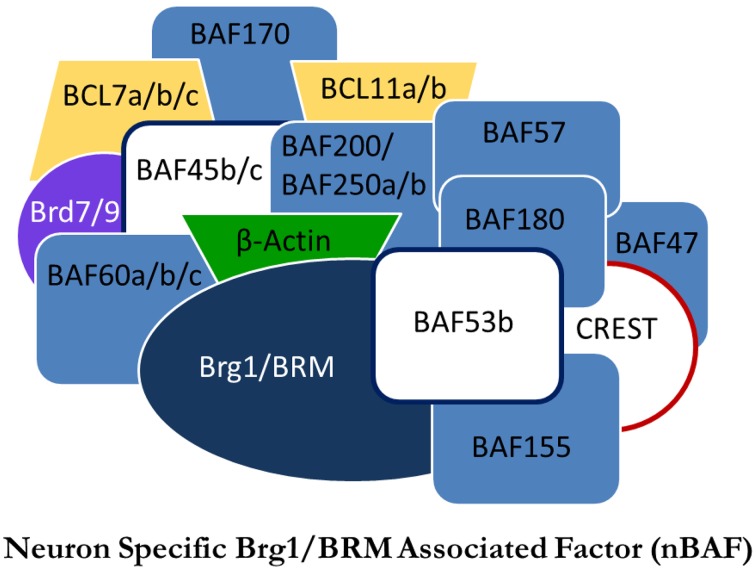
**Proposed model of the nBAF complex**. Subunits in white are subunits thought to be neuron-specific. Adapted from Staahl and Crabtree (2013).

**Figure 2:**
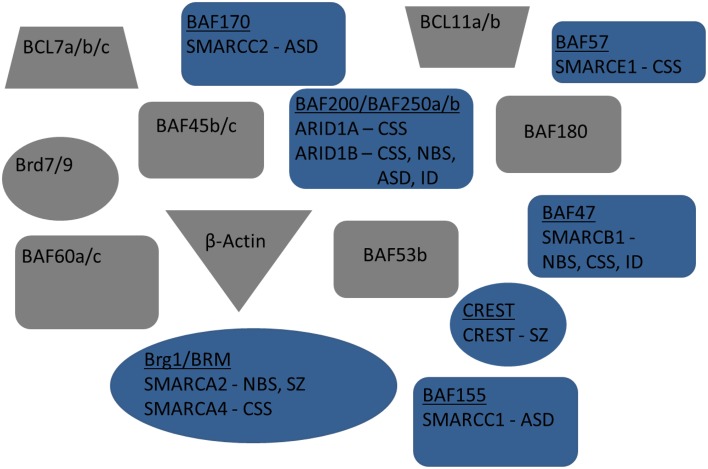
**Exploded view of nBAF complex**. Mammalian subunits associated with neuro developmental and/or cognitive disorders are in blue. Human gene name is also included below subunit name. Mammalian subunits in gray have no currently known mutations linked with new:odevelopment or adult cognition.

NRCs, particularly SWI/SNF, are well conserved throughout mammalian and human cells (Table 1, Figure 1). The human homologs of the yeast NRCs have similar critical roles in regulating functional and developmental gene expression in higher order mammals, including humans. Recently, research efforts have examined the human genome, through both genome-wide association studies and human exome sequencing. These new sequencing efforts have led to the discovery of several mutations in genes coding for nBAF subunits that are believed to be causal mechanisms giving rise to various ID disorders, ASD, and other developmental disorders. The majority of mutations have been found in the *SMARC* and *ARID* families of genes, which code proteins of the nBAF complex (Hargreaves and Crabtree, 2011; Wilson and Roberts, 2011; Santen et al., 2012b, 2013). SMARC proteins are known to have helicase and ATPase activity and are thought to be critical in nucleosome remodeling. In contrast, ARID proteins have DNA recognition binding sites and are thought to give BAF complexes gene specificity. Coffin-Siris Syndrome (CSS), an ID disorder, may be caused by various mutations in *ARID* and *SMARC* genes (Table 1, Figure 2). First reported in 1970 by Drs. Coffin and Siris, CSS is characterized by ID and joint abnormality, particularly in the fifth digit (Coffin and Siris, 1970). Mutations in *ARID1B* have been found in patients showing agenesis of corpus callosum, intellectual disorder, speech impairment, and varying degrees of autism severity (Halgren et al., 2012). *ARID1B* mutations have also been found in several patients diagnosed with CSS, along with mutations in *ARID1A* and several *SMARC* genes including *SMARCA4*, *SMARCA2, SMARCE1*, and *SMARCB1* (Santen et al., 2012a; Tsurusaki et al., 2012; Parikshak et al., 2013; Miyake et al., 2014).

**Table 1:** **BAF subunit coding genes implicated in neurological disorders**.

**Gene family**	**SWI/SNF homolog**	**Gene**	**Associated disorder**	**References**
*ARID*	SWI1	ARID1A	css	Staahl and Crabtree, 2013
		ARID1B	CSS, NBS, ID, ASD	Backx et al., 2011; Nord et al., 2011; Halgren et al., 2012; Hoyer et al., 2012
*SMARC*	SWI2, ISNF2	SMARCA2	NBS, SZ	Koga et al., 2009; Van Houdt et al., 2012; Wolff et al., 2012
		SMARCA4	css	Tsurusaki et al., 2012, 2014; Staahl and Crabtree, 2013
		SMARCB1	NBS, CSS, ID	Santen et al., 2012a,b, 2013; Tsurusaki et al., 2012, 2014; Staahl and Crabtree, 2013; Miyake et al., 2014
	SWI3	SMARCC1	ASD	Neale et al., 2012
		SMARCC2	ASD	Neale et al., 2012
		SMARCE1	css	Tsurusaki et al., 2012, 2014
*CREST*		CREST	sz	Chesi et al., 2013

*Adapted from and Staahl and Crabtree (2013) and Collingwood et al. (1999). ASD, Austism Spectral Disorder; CSS, Coffin-Siris Syndrome; ID, Intellectual Disability; NBS, Nicolaides-Baraitser Syndrome; SZ, Schizophrenia*.

Several BAF complex genes have also been implicated with Nicolaides-Baraitser syndrome (NBS) (Table 1, Figure 2). Patients with NBS are characterized by severe mental retardation, seizures, and limited speech (Nicolaides and Baraitser, 1993). Sequencing studies have discovered mutations in *SMARCA2* in NBS patients and patients with intellectual disorders, along with mutations in *ARID1B* and *SMARCB1* (Van Houdt et al., 2012; Santen et al., 2013; Miyake et al., 2014). Additionally, mutations have been discovered in *ADNP*. Although not considered a subunit of the BAF complex, ADNP is known to interact with several of the core BAF subunits, such as SMARCA4, SMARCC2, and ARID1A (Mandel and Gozes, 2007). These discovered mutations are thought to be causally related to development of CSS and perhaps even de novo mutations in ASD (Ben-David and Shifman, 2012; Helsmoortel et al., 2014; Vandeweyer et al., 2014). Although the same genes (e.g., *SMARC* genes) are implicated in different ID disorders, this is likely due to when during development the mutation occurred, as well as cell type-specific effects. A main characteristic of NRCs is the numerous subunits, including cell type-specific subunits (as observed in the neuron-specific nBAF genes described below), which give rise to combinations that are developmental stage-specific and cell type-specific. This is also quite likely why similar complexes have roles in the adult brain related to cognition that are independent from their developmental roles.

For example, research on CBP has demonstrated a clear role for CBP in development as well as adult brain cognitive processes. There are debates in the literature whether RTS was primarily caused by loss of one allele of *CBP*, leading to a heterozygous condition (Tanaka et al., 1997), or mutations and deletions giving rise to dominant negative alleles of *CBP* (Oike et al., 1999; see Barrett and Wood, 2008 for review). Although that debate continues, researchers have been able to address a different question: whether CBP has a role in adult cognition that is independent of its role in development. This is an important question because if CBP does have an independent role in adult cognition, it may be possible one day to treat cognitive impairments associated with mutations in CBP. Three early studies addressed this question by demonstrating that indeed CBP has a role in adult cognition, specifically with regard to long-lasting forms of synaptic plasticity and long-term memory formation (Alarcón et al., 2004; Korzus et al., 2004; Wood et al., 2005; Barrett et al., 2011). One idea to ameliorate cognitive impairments in genetically modified *Cbp* mutant mice was to use HDAC inhibitors, which would effectively increase histone acetylation. Simply blocking HDAC activity shouldn't *a priori* result in an increase in histone acetylation, yet that is what is observed in nearly all studies (reviewed in McQuown and Wood, 2011), suggesting that there is a dynamic interplay between HDACs and HATs. Thus, removal of HDAC activity allows for HATs to engage and increase histone acetylation. Interestingly, in some forms of synaptic plasticity and memory, HDAC inhibition enhances memory in a CBP-dependent manner, which brings into question the ability to use HDAC inhibition as a blanket treatment for RTS patients (Vecsey et al., 2007; Barrett et al., 2011; Haettig et al., 2011).

## BAF complexes in neural development

The SWI/SNF family of nucleosome remodelers are well conserved from yeast to mammals. BRG1/Brm-associated factor (BAF), homologous to SWI/SNF in yeast and brahma in drosophila, is one such complex. In contrast to the SWI/SNF complex, the specific functions of the BAF complex remain unknown, yet are presumed to be similar. The various mechanisms of nucleosome remodeling employed by NRCs have been thoroughly reviewed (Li et al., 2007; Bartholomew, 2014). Although the nucleosome remodeling functions of BAF remain unclear, BAF has been shown to be critical in cellular development, particularly in regulated gene expression throughout mitosis. With regards to mammalian development, there is a neuron-specific NRC with functional roles in both neural development and adult cognition. Neuron-specific BAF (nBAF) has three dedicated subunits differentiating it from BAF: BAF53b, BAF45b, and BAF45c (Figure 1). The exchange of these subunits with their non-neuronal analogs is a critical switch in neural development and differentiates neural progenitor BAF from nBAF (Olave et al., 2002; Hargreaves and Crabtree, 2011). Of particular importance is the dedicated neuron-specific subunit BAF53b. BAF53b expression begins at E12.5 and is exclusive to post-mitotic neurons; it is not found in other epidermal tissue (Olave et al., 2002; Bao et al., 2012). It has been shown that the regulatory switch of upregulating BAF53b, BAF45b, and BAF45c, while repressing expression of BAF53a, is critical in establishing neural cell fate and is the final step in generating post-mitotic neurons (Olave et al., 2002; Lessard et al., 2007; Hargreaves and Crabtree, 2011). When BAF53b expression is inhibited, or, conversely, when BAF53a expression is maintained, neural differentiation is prevented (Hargreaves and Crabtree, 2011). The conversion from BAF53a to BAF53b is a vital switch for neuronal development that has critical implications for adult cognition.

Functionally, BAF53b has been shown to associate with BRG1, both *in vivo* and *in vitro*, and is necessary for BRG1's ATPase function in neurons (Zhao et al., 1998; Olave et al., 2002). Cultured neurons with BAF53b deletions show a loss of activity-dependent dendritic growth that is restored with exogenous expression of BAF53b. Moreover, deletions of *Baf53b* in mice are lethal (Wu et al., 2007). However, BAF53b is not required for the complete formation of the nBAF complex and does not have independent ATPase function (Zhao et al., 1998; Olave et al., 2002; Wu et al., 2007). This suggests that BAF53b may not directly alter nBAF's enzymatic activity, but as an actin-related protein, may serve a scaffolding function for the recruitment of additional subunits that help target the complex to specific gene promoters. One such subunit is the calcium-responsive transactivator (CREST). CREST is known to regulate dendritic arborization and form a neuron-specific complex with nBAF (Aizawa, 2004; Wu et al., 2007). Related work has shown that BAF53b is necessary for the recruitment of the nBAF/CREST complex to particular gene promoters, including *Ephexin1*, a GTPase critical for synapse remodeling and maturation (Wu et al., 2007; Shi et al., 2010a,b). Lastly, nBAF has been shown to have a role in neural subtype specificity. For example, the nBAF complex is known to regulate *Sem-4*. SEM-4 consists of various zinc fingers and has been shown to be required for both neuronal and mesodermal cellular development. The zinc-fingers are thought to differentiate SEM-4's roles in neural development from its roles in mesodermal development (Basson and Horvitz, 1996). When *Ham-3* specific mutations are introduced, loss of BAF-dependent control of *Sem*-4 leads to dysregulation of serotonergic neuronal cell fate (Weinberg et al., 2013). HAM-3, a Striatin homolog, is part of the STRIPAK complex needed for MAP kinase regulation (Dettmann et al., 2013; Hwang and Pallas, 2014). The above research indicates that nBAF has a role in initiating neural differentiation and regulating neural development. What about adult cognition?

## BAF complexes in cognition

The next questions are what makes BAF53b functionally unique from its progenitor analog, BAF53a, do these functional differences give rise to selective gene expression, and are the roles of these discretely regulated genes in development (which mutations give rise to developmental disorders) distinguishable from their roles in adult cognition. We recently published a study that used two different genetically modified BAF53b mutant mice that allowed us to begin addressing these questions (Vogel-Ciernia et al., 2013). One transgenic animal was generated to target the hydrophobic domain, responsible for protein-protein interactions, of BAF53b (*BAF*53bΔHD mice). Deletions of the hydrophobic domain in BAF53a are known to generate dominant-negative forms of BAF53a (Park et al., 2002) and predicted to have a similar effect in BAF53b. The other animal was a conventional heterozygous mouse *BAF*53b^+/−^ mice; generated by Dr. Gerald Crabtree's lab, (Wu et al., 2007)). Both *BAF*53b^+/−^ and *BAF*53bΔHD animals exhibited large impairments in long-term memory formation in the object location memory (OLM) task (Vogel-Ciernia et al., 2013). This deficit was rescued in BAF53b^+/−^ by acutely restoring expression of BAF53b to the dorsal hippocampus, a region known to be necessary for the OLM task (Stefanko et al., 2009; Haettig et al., 2011; McQuown et al., 2011; Vogel-Ciernia et al., 2013). Together, these results suggest that BAF53b is necessary and sufficient for the formation of long-term memory.

Importantly, the hydrophobic domain is not a unique subdomain to differentiate BAF53a from BAF53b. Although BAF53b and BAF53a are structurally similar, the most divergent region is subdomain 2. Subdomain 2 is required for the BAF53b-dependent dendritic outgrowth seen in cultured neurons (Wu et al., 2007). To evaluate the role of BAF53b's Subdomain 2 and how it differs from BAF53a's Subdomain 2, Wu et al. (2007) created chimeric versions of BAF53a and BAF53b, interchanging their respective Subdomain 2. The chimeric BAF53a (containing BAF53b's Subdomain 2) was able to restore the dendritic branching of BAF53b^−/−^ neuronal cultures, while also restoring BAF53b-dependent expression of *Ephexin* and *Gap43*. However, the inverse chimera (BAF53b containing BAF53a's Subdomain 2) was unable to rescue the loss of dendritic growth and gene expression (Wu et al., 2007). These critical experiments show that it is Subdomain 2 of BAF53b that is necessary for neuronal development and neuron-specific gene expression. Thus, Subdomain 2 is the key domain that differentiates the function of BAF53b from the function of BAF53a. Additionally, BAF53b is one of the neuron-specific subunits of nBAF, and BAF53b is a dedicated subunit of the nBAF complex (thus not found in other complexes, as far as the field understands at this point), which makes the BAF53b Subdomain 2 an ideal target to study the neuronal function of BAF53b in adult synaptic plasticity and memory formation. It will be very important to understand the role of BAF53b Subdomain 2 in cognition and also determine whether Subdomain 2 has unique protein-protein interactions, phosphorylation sites, etc. Subdomain 2 may provide critical insight into understanding the neuron-specific role of nBAF in adult cognition.

## Enhanceosome

As previously discussed, it is critical to understand the dynamics of these gene regulatory mechanisms. In order to do so, we must understand that these epigenetic mechanisms occur, both spatially and temporally, in conjunction with each other and other gene regulatory elements. One element, which also recruits protein complexes to control gene expression, is the enhanceosome. Enhanceosomes refer to the transcription factor complexes that assemble and bind to gene enhancers and recruit other modifying enzymes (Thanos and Maniatis, 1995; Merika and Thanos, 2001; Panne, 2008). The assembly of particular transcription factors in such a manner allows for a gene-specific level of regulation. One well-studied enhanceosome system regulates interferon-β (*I*fnβ) gene expression. The chromatin surrounding *I*fnβ is typically compressed, while its enhancer element remains exposed between nucleosomes. *I*fnβ expression is relatively suppressed; upon exposure to viral infection, its expression dramatically increases through the assembly of a particular enhanceosome complex to the *I*fnβ enhancer. This complex is able to selectively recruit histone acetyltransferases (such as CBP and p300) to nearby chromatin, leading to histone acetylation and SWI/SNF complex recruitment (Merika et al., 1998; Yie et al., 1999; Agalioti et al., 2000; Koutroubas et al., 2008).

This form of gene regulation is not unique to *I*fnβ expression. Several eukaryotic genes have been shown to be regulated in a similar fashion, including *Elam-1*, *Hmg-1*, *Interleukin-6*, and *Interleukin-2* (Whitley et al., 1994; John et al., 1995; Vanden Berghe et al., 1999; Ellwood et al., 2000; Merika and Thanos, 2001). The dynamic and conjunctive activity of these enzymes, activating NRCs, generate a more permissive state allowing basic transcription factors access to the respective genes of interest (Kim and Maniatis, 1997; Agalioti et al., 2000; Merika and Thanos, 2001). Yet, enhanceosome-mediated gene regulation in the nervous system has received relatively little attention. With the crucial role nucleosome remodeling complexes have in proper nervous system function, enhanceosomes may be as important in regulating the proper gene expression in processes from neural development to long-term memory formation.

## Conclusion

In this review we have focused on the role of nucleosome remodeling in development and adult cognition. Several human sequencing studies have shown that mutations in the genes making up the BAF complex in particular may give rise to distinct ID disorders including CSS, NBS, and ASD. These human studies highlight how critically important nucleosome remodeling is for proper development and cognitive function. One central theme we discussed was the role of nucleosome remodeling in development and whether that role is independent of a role for nucleosome remodeling in adult cognition. That question can only be understood by studying the role of nucleosome remodeling in model organisms. Indeed, research has demonstrated for several epigenetic regulatory enzymes (e.g., CBP) and DNA methylation binding proteins (e.g., MeCP2) and more recently for nucleosome remodeling (e.g., BAF53b), that these factors have a role in adult neuronal plasticity, learning, and memory, independent of their developmental roles. It remains difficult to prove that this is also the case in adult ID disorders associated with mutations in these genes, but it gives hope that cognitive dysfunction observed in these ID disorders may one day be at least partially ameliorated.

### Conflict of interest statement

The authors declare that the research was conducted in the absence of any commercial or financial relationships that could be construed as a potential conflict of interest.
